# Genomic evaluation of phenotypic antibiotic susceptibility patterns as a surrogate for MRSA relatedness and putative transmission during outbreak investigations

**DOI:** 10.1016/j.infpip.2024.100435

**Published:** 2024-12-26

**Authors:** Francesc Coll, Michelle S. Toleman, Ewan M. Harrison, Beth Blane, Dorota Jamrozy, Nicholas M. Brown, Julian Parkhill, Sharon J. Peacock

**Affiliations:** a Applied Microbial Genomics Unit, Department of Molecular Basis of Disease, Institute of Biomedicine of Valencia (IBV-CSIC), Valencia, Spain; b Department of Infection Biology, Faculty of Infectious and Tropical Diseases, London School of Hygiene & Tropical Medicine, Keppel Street, London WC1E 7HT, UK; c Wellcome Sanger Institute, Hinxton, Cambridge, UK; d Clinical Microbiology and Public Health Laboratory, UK Health Security Agency, Cambridge CB2 0QQ, UK; e Department of Medicine, University of Cambridge, Box 157 Addenbrooke's Hospital, Hills Road, Cambridge, CB2 0QQ, UK; f Department of Veterinary Medicine, University of Cambridge, Cambridge CB3 0ES, UK

## Abstract

Antibiograms have been used during outbreak investigations for decades as a surrogate for genetic relatedness of Methicillin-resistant *Staphylococcus aureus* (MRSA). In this study, we evaluate the accuracy of antibiograms in detecting transmission, using genomic epidemiology as the reference standard. We analysed epidemiological and genomic data from 1,465 patients and 1,465 MRSA isolates collected at a single clinical microbiology laboratory in the United Kingdom over a one-year period. A total of 132 unique antibiograms (AB) were identified based on VITEK 2 susceptibility testing, with two profiles (AB1 and AB2) accounting for 698 isolates (48%). We identified MRSA-positive patients with a known hospital or community contact and evaluated the prediction of MRSA transmission based on identical antibiograms. The sensitivity and specificity of identical antibiograms to infer genetically related MRSA isolates (≤25 SNPs) within hospital contacts (presumed transmission events) was 66.4% and 85.5% respectively and 73.8% and 85.7% within community contacts. Reanalysis, where any single drug mismatch in susceptibility results was allowed, increased sensitivity but reduced specificity: 95.2% and 58.8%, respectively, for hospital contacts; and 91.7% and 62.6% for community contacts. Overall, the sensitivity and specificity of identical antibiograms for inferring genetically related MRSA isolates (≤25 SNPs), regardless of epidemiological links, were 49.1% and 87.5%, respectively. We conclude that using an antibiogram with one mismatch can detect most transmission events; however, its poor specificity may lead to an increased workload through the evaluation of numerous pseudo-outbreaks. This study further supports the integration of genomic epidemiology into routine practice for the detection and control of MRSA transmission.

## Importance

The antimicrobial susceptibility and resistance pattern (or antibiogram) is commonly used as a surrogate for genetic relatedness during the evaluation of putative transmission and outbreaks of MRSA. This approach is supported by the ready availability of susceptibility data from routine microbiological testing. Whole genome sequencing has highlighted the limitations of this practice, but there is a lack of systematic evidence quantifying its accuracy. To address this, we analysed data from a cohort of 1,465 MRSA-positive patients for whom MRSA genome and epidemiological information were available. Our findings show that the sensitivity and specificity of an identical antibiogram in predicting MRSA transmission were low. Sensitivity improved when allowing for a single drug mismatch in susceptibility, but at the cost of specificity. These results provide further support for the introduction of genomic epidemiology into routine practice for the detection and management of MRSA transmission.

## Introduction

Infection prevention and control (IPC) teams monitor the isolation of Methicillin-resistant *Staphylococcus aureus* (MRSA) in healthcare settings to detect clusters of positive cases in time and space. Apparent clusters are investigated to evaluate the possibility of an outbreak, which may include bacterial typing to determine the genetic relatedness of the isolates, which helps infer the likelihood of transmission. Genome sequencing provides the highest resolution for differentiating MRSA isolates and has been shown to enhance outbreak investigation and management [[1], [2], [3], [4], [5]]. However, this technology is not currently available in routine practice, and other, less discriminatory but more readily available data may be used.

For decades, IPC practitioners have used the pattern of antimicrobial susceptibility and resistance (referred to as the ‘antibiogram’) as a surrogate marker for the genetic relatedness of MRSA isolates. Antibiotic susceptibility data are generated as part of routine microbiological processes and provide readily available information at the outset of an outbreak investigation. However, the accuracy of the antibiogram when compared to a gold standard, such as genomic data, is poorly defined. Additionally, since nosocomial MRSA outbreaks are generally caused by a limited number of genetic lineages [[1], [2], [3], [4], [5]], antibiograms may have limited utility in confirming an outbreak or distinguishing between true outbreaks and pseudo-outbreaks, especially if antibiograms are similar across specific lineages. On the other hand, antibiograms may vary between MRSA isolates that are genetically highly related [2], which could lead to the erroneous exclusion of cases from a cluster. This is of central importance to IPC practice. Declaring an MRSA outbreak, whether true or not, is a significant event that may lead to interventions being put in place to control its spread, such as the closure of parts or all of a clinical area. By contrast, erroneously excluding a link between MRSA isolates can lead to harm by allowing further transmission and delaying recognition of the outbreak.

In this study, we evaluate the sensitivity and specificity of the phenotypic antibiogram, used in combination with patient movement data, to predict MRSA transmission. These predictions were compared against genomic epidemiology data (including MRSA genomes and patient movement) as the reference standard.

## Materials and methods

### Ethical approval, study setting and patients

The study was based on data gathered during a 12-month prospective observational cohort study conducted at a hospital in the East of England between April 2012 and April 2013, which has been reported previously [4]. In brief, 1,465 consecutive individuals with MRSA-positive samples were identified by the Clinical Microbiology and Public Health Laboratory at the Cambridge University Hospitals NHS Foundation Trust (CUH). Samples were submitted by three hospitals and 75 general practitioner (GP) surgeries. Cases had a median age of 68 years [range newborn to 101 years, interquartile range (IQR): 46–82 years]. Epidemiological data on inpatient hospital stays and residential postcodes were recorded for all cases, from which links were sought between every pair of MRSA-positive individuals (case-pairs), as described previously [4]. For the purposes of this study, we defined an epidemiological link if a case-pair had a direct ward contact (i.e., admitted to the same ward with overlapping dates of admission for at least 24 hours) or if cases shared a postcode or GP practice (i.e., community contact as defined previously [4]). In all cases, we sought details of any hospital admissions and ward transfers during the 12-month period prior to their first MRSA-positive sample. Ethical approval for the study was provided by the National Research Ethics Service (ref: 11/EE/0499), the National Information Governance Board Ethics and Confidentiality Committee and the Cambridge University Hospitals NHS Foundation Trust Research and Development Department.

### Microbiology, antibiograms and genomic analyses

MRSA was isolated from screening swabs and clinical samples as previously described [4]. The original study included 2,282 MRSA isolates from 1,465 patients after genomic quality control; however, for this analysis, we excluded consecutive samples from the same individual and focused on their first MRSA isolate (1,465 isolates from 1,465 patients). The antimicrobial susceptibility of each isolate was determined using the VITEK 2 instrument (bioMérieux, Marcy l’Etoile, France) with the European Committee on Antimicrobial Susceptibility Testing (EUCAST) breakpoints and the AST-P620 card. The antibiotics tested included benzylpenicillin, cefoxitin, oxacillin, ciprofloxacin, erythromycin, chloramphenicol, daptomycin, fusidic acid, gentamicin, linezolid, mupirocin, nitrofurantoin, rifampicin, teicoplanin, tetracycline, tigecycline, trimethoprim, vancomycin, and clindamycin. For this analysis, susceptibility results reported as ‘intermediate’ were categorised as resistant. The susceptibility and resistance patterns for these 19 antibiotics was compared between every isolate-pair (1,072,380 pairwise comparisons), and each unique antibiogram was labelled sequentially as AB1, AB2, AB3 etc., with AB1 representing the most frequent antibiogram, AB2 the second most frequent, and so on.

We defined two criteria for a “highly similarity antibiogram” between MRSA isolates based on real-world IPC practices used to infer MRSA relatedness and the likelihood of transmission: (i) an isolate pair had an identical antibiogram, or (ii) an isolate-pair had at most one mismatch in their susceptibility patterns (involving any one of the antibiotics tested). Differences involving two or more mismatches were not considered.

Sequence data for the 1,465 MRSA isolates were downloaded from the European Nucleotide Archive (ENA) under the PRJEB3174 study accession (Supplementary Table 1). Multilocus sequence types (STs) were derived from de novo assemblies using mlst_check (https://github.com/sanger-pathogens/mlst_check). Clonal complexes (CCs) were assigned based on the MLST allelic profile, allowing up to two allele mismatches from the reference ST. We analysed the relationship between clonal complexes and antibiograms by identifying the unique antibiograms present within each CC. We also determined the genetic relatedness between isolates within each clonal complex by comparing the number of single nucleotide polymorphisms (SNPs) in their whole genomes after masking mobile genetic elements. This comparison was performed in a pair-wise manner. Isolates were considered ‘genetically related’ if the SNP difference between two isolates was ≤25 SNPs, as previously evaluated and proposed^6^.

### Accuracy of antibiograms to identify MRSA transmission

We assessed the accuracy of the antibiogram, combined with epidemiological link data, in confirming or refuting MRSA transmission, using genomic epidemiology as the reference standard. MRSA transmission was defined between case-pairs when there was a known epidemiological link and their isolate genomes were genetically related. MRSA transmission was refuted if an epidemiological link existed between case-pairs, but their MRSA isolate genomes exceeded the SNP cut-off for relatedness (pseudo-transmission). We calculated the sensitivity, specificity, and predictive values of (i) an identical antibiogram, and (ii) an antibiogram with one mismatch using the following formulas:

•Sensitivity=[TP/(TP+FN)]×100

•Specificity=[TN/(TN+FP)]×100

•PositivePredictiveValue(PPV)=[TP/(TP+FP)]×100

•NegativePredictiveValue(NPV)=[TN/(FN+TN)]×100

where TP = true positive, FP = false positive, TN = true negative and FN = false negative. The analysis was performed twice, for transmission events in hospital wards (direct ward link) and in the community (shared postcode or GP visit).

### Accuracy of antibiograms to infer MRSA relatedness

We assessed the accuracy of the antibiogram as a surrogate marker of MRSA relatedness, which is the rationale for using antibiogram patterns during outbreak investigations. For this analysis, we considered all pairwise comparisons of MRSA isolates, regardless of the presence of an epidemiological link, using the whole genome ≤25 SNP cut-off as the reference benchmark. As before, we calculated the sensitivity, specificity, and predictive values of (i) an identical antibiogram, and (ii) an antibiogram with one mismatch. Additionally, we conducted a sensitivity analysis using different genetic relatedness thresholds (15 [7], 25 [6] and 50 [4] SNPs, all of which have been proposed previously).

### Accuracy of antibiogram after excluding the two most common antibiograms

Two antibiogram profiles (AB1 and AB2) accounted for nearly half of the MRSA isolates (see results). A perception among some IPC practitioners is that less frequent or unusual antibiograms may be more likely to indicate MRSA relatedness and predict MRSA transmission. To test this hypothesis, we removed isolates with AB1 and AB2 antibiograms and re-ran the analysis. As before, we calculated the sensitivity, specificity, and predictive values of (i) an identical antibiogram, and (ii) an antibiogram with one mismatch.

## Results

We utilised an epidemiological and genomic dataset collected on 1,465 patients and MRSA isolates over a one-year period. This previously published integrated epidemiological and phylogenetic analysis revealed 173 transmission clusters, each containing between 2 and 44 cases and involving 598 people (40.8%). Of these, 118 clusters (371 people) involved hospital contacts alone, 27 clusters (72 people) involved community contacts alone, and 28 clusters (157 people) involved both types of contact [4]. This unbiased, consecutive patient/isolate dataset provided the foundation for determining the antibiogram and evaluating its utility in routine IPC practice for investigating putative MRSA outbreaks.

**Figure 1:**
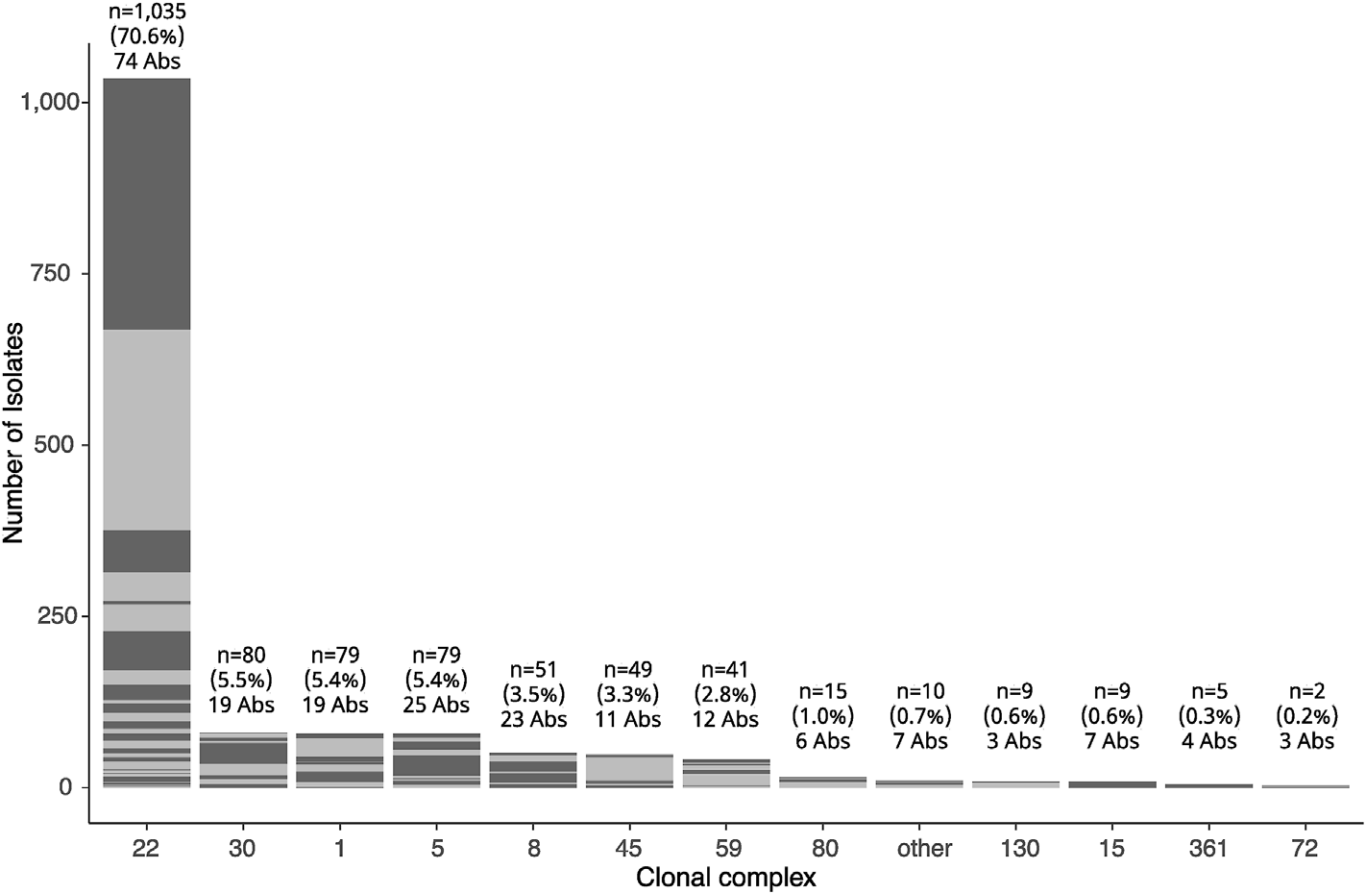
Diversity of antibiograms within different MRSA clonal complexes. Bar plot displaying the diversity of unique antibiograms within different clonal complexes identified among 1,465 MRSA isolates. Unique antibiograms (n=132) are displayed by alternating two shades of gray so that each segment represents a single antibiogram. Clonal complexes (CCs) are ordered in decreasing frequency on the x-axis, with rarer CCs (identified in less than three isolates) labelled as “other”, including information on total number of MRSA isolates, percentage of isolates, and number of unique antibiograms (Abs) per CC.

Testing the susceptibility of 1,465 isolates against 19 antibiotics using the VITEK 2 instrument revealed a total of 132 unique antibiograms, although two profiles (AB1 and AB2) accounted for 698 (48%) of isolates (Supplementary Table 1). Sequence types derived from sequencing data indicated a genetically diverse collection, with 58 STs belonging to 19 CCs. However, the collection was dominated by CC22 (1,035 isolates, 71%) (Figure 1). The number of different antibiograms was determined for each CC, revealing a high degree of antibiogram diversity within the same CC (Figure 1). For example, CC22 contained 74 distinct antibiograms (Supplementary Table 1).

We evaluated the prediction of MRSA transmission based on antibiogram patterns for individuals with direct hospital ward contact (3,104 case-pairs, 1,001 patients), of which 505 case-pairs (421 patients) had MRSA isolates with identical antibiograms. Unblinding of genetic data showed the sensitivity and specificity of an identical antibiogram to predict MRSA transmission were 66.4% and 85.5%, respectively, with a positive predictive value (PPV) of 13.7% within this cohort (Table I). We repeated the analysis with a more relaxed definition of an antibiogram, allowing for up to one mismatch in susceptibility (1,336 case-pairs). This increased the sensitivity at the cost of specificity (95.2% and 58.8%, respectively) (Table I). Given that nearly half of MRSA isolates (n=698, 48%) had one of two antibiograms (AB1 and AB2), we re-analysed a subset of the data after excluding AB1 and AB2 isolates. This aimed to determine whether rarer antibiograms would lead to greater accuracy in detecting or excluding putative transmission. The resulting sensitivity and specificity for an identical antibiogram to detect a direct ward contact was 64.6% and 97.2%, respectively (Table I).

**Table I:** Sensitivity, specificity, and predictive values of the antibiogram to identify MRSA transmission between different categories of epidemiologically related isolate pairs in our cohort

Combination									
***Hospital Epidemiological link (Same ward, Same time)***	No. of comparisons	TP	FP	TN	FN	Sensitivity (%)	Specificity (%)	PPV (%)	NPV (%)

Identical antibiogram (n=505 case-pairs)	3,104	69	436	2,564	35	66.4	85.5	13.7	98.7
Single mismatch in antibiogram (n=1,336 case-pairs)	3,104	99	1,237	1,763	5	95.2	58.8	7.4	99.7
Identical antibiogram excluding pattern (AB) 1 and 2 (n=72 case-pairs)	836	51	21	736	28	64.6	97.2	70.8	96.3
***Community Epidemiological link (Same Postcode)***									
Identical antibiogram (n=112 case-pairs)	434	62	50	300	22	73.8	85.7	55.4	93.2
Single mismatch in antibiogram (n=208 case-pairs)	434	77	131	219	7	91.7	62.6	37.0	96.9
Identical antibiogram excluding pattern (AB) 1 and 2 (n=44)	133	35	9	82	7	83.3	90.1	79.5	92.1

Abbreviations: AB, antibiogram; TP, true positive; FP, false positive; TN, true negative; FN, false negative; PPV, positive predictive value; NPV, negative predictive value.

Coll *et al.* showed that a shared postcode is a strong epidemiological link for putative MRSA transmission [4]. Therefore, we assessed the accuracy of antibiograms in predicting transmission between case-pairs with a shared residential postcode (n=434). Of these, 112 case-pairs (146 patients) had isolates with an identical antibiogram. Comparison with genome data demonstrated a sensitivity and specificity of 73.8% and 85.7%, respectively (Table I). Using the antibiogram with one mismatch, the sensitivity and specificity were 91.7% and 62.6%, respectively (Table I). Analysis of the subset excluding the two dominant antibiograms (AB1 & AB2) resulted in a sensitivity and specificity for a matching antibiogram of 83.3% and 90.1%, respectively.

Next, we assessed the accuracy of identical antibiograms to infer genetic relatedness of MRSA isolates, independent of the presence and type of epidemiological link (Table II), using different genetic relatedness thresholds (15, 25 and 50 SNPs). The sensitivity of identical antibiograms to detect genetically related isolates was modest (ranging from 39.6 to 55.1%, depending on the SNP threshold) and fell with higher SNP thresholds. As expected, one-mismatch antibiograms showed higher sensitivity at the expense of lower specificity, and the exclusion of the two most common antibiogram patterns improved specificity (Table II). Predictably, PPVs were low due to the large number of false positives (pairs of isolates with matching antibiograms but not genetically related). However, PPV's improved once the two common antibiogram patterns were excluded.

**Table II:** Sensitivity, specificity, and predictive values of the antibiogram to identify MRSA genetic relatedness using different genetic relatedness thresholds

Combination									
***Genetic link ( ≤ 15 SNPs)***	No. Comparisons^a^	TP	FP	TN	FN	Sensitivity (%)	Specificity (%)	PPV (%)	NPV (%)

Identical antibiogram	1,072,380	685	133,920	937,216	559	55.1	87.5	0.5	99.9
Single mismatch in antibiogram	1,072,380	1,137	422,351	648,785	107	91.4	60.6	0.3	100.0
Identical antibiogram excluding pattern (AB) 1 and 2	293,761	511	11,486	281,302	462	52.5	96.1	4.3	99.8
***Genetic link (*** ≤ ***25 SNPs)***									
Identical antibiogram	1,072,380	1,232	133,373	936,497	1,278	49.1	87.5	0.9	99.9
Single mismatch in antibiogram	1,072,380	2,258	421,230	648,640	252	90.0	60.6	0.5	100.0
Identical antibiogram excluding pattern (AB) 1 and 2	293,761	975	11,022	280,690	1,074	47.6	96.2	8.1	99.6
***Genetic link (*** ≤ ***50 SNPs)***									
Identical antibiogram	1,072,380	2,283	132,322	934,298	3,477	39.6	87.6	1.7	99.6
Single mismatch in antibiogram	1,072,380	4,767	418,721	647,899	993	82.8	60.7	1.1	99.8
Identical antibiogram excluding pattern (AB) 1 and 2	293,761	1,341	10,656	280,044	1,720	43.8	96.3	11.2	99.4

a Considering all possible pairwise comparisons for all 1,465 MRSA patients, regardless of type of epidemiological link between them. After excluding the two most common antibiograms, 767 MRSA patients (293,761 pairwise comparisons) were left.

## Discussion

Antibiograms are frequently used in combination with patient movement data as indicators of isolate relatedness and potential MRSA transmission events. However, studies using genomic epidemiology have suggested that they can be inaccurate. For example, a study of an MRSA outbreak in a special care baby unit reported that several early isolates, initially excluded based on their antibiogram, were later found to be part of the outbreak through genome sequencing [2]. Similarly, a study of 20 *S. aureus* outbreaks reported that isolates in five of 17 MRSA outbreaks contained two or more antibiograms despite genetic relatedness [8]. However, a systematic population-based comparison of antibiogram against genome sequencing has not been reported until now.

In our large cohort study on MRSA transmission, using whole genome sequence data as the gold standard method for defining isolate relatedness, we found that antibiogram plus epidemiology had low sensitivity and specificity for identifying MRSA transmission in hospital settings. Removing the two most common antibiogram patterns yielded similar results, indicating that less common antibiogram patterns do not improve the accuracy of predicting transmission. The sensitivity of the antibiogram improved when one mismatch was allowed but this resulted in a lower specificity. Due to the low prevalence of genetically related MRSA isolates in this cohort, the positive predictive value of all analyses was very low, suggesting that IPC interventions based on epidemiological links and antibiogram patterns are insufficiently targeted to true outbreaks in such cohorts. However, the negative predictive value of the antibiogram combined with epidemiological data was considerably higher. A smaller analysis of community transmission using antibiograms and shared postcodes demonstrated sensitivities and specificities that were not sufficiently high to be useful in practice.

This study has several limitations. Antibiograms were determined using the VITEK 2 instrument, which provides information on 19 antibiotics, but this may not be available for all isolates in every clinical laboratory. Alternative methods, such as disc diffusion, are commonly used but typically generate smaller datasets (e.g., susceptibility data for 6 or 12 antibiotics), which may be less discriminatory and could further impact sensitivity, specificity and PPVs. Additionally, accuracy measures such as PPV and NPV are highly dependent on prevalence and could vary in different cohorts. However, this does not detract from our conclusion that antibiograms were unreliable for determining transmission events. Another limitation of our study, and of using antibiograms more broadly, is the potential for misclassification of antibiotic susceptibility testing (AST). While the error rate for AST in *S. aureus* is low [9], and use of VITEK 2 is reflective of clinical practice in many laboratories, this remains a consideration.

Despite its limitations, the study provides valuable insights into the accuracy of epidemiology combined with antibiograms for defining putative MRSA transmission in this large cohort. Our systematic analysis demonstrates that, compared to the gold standard of sequencing plus epidemiology, the antibiogram is not an accurate method for inferring MRSA relatedness and does not reliably identify or refute transmission events. These findings underscore the need for MRSA sequencing in routine practice to reliably confirm or refute transmission and outbreaks.

## Transparency declarations

SJP and JP are consultants to Next Gen Diagnostics. The remaining authors declare no conflict of interest.

## Appendix A Supplementary data

Supplementary table 1 Details of MRSA isolates used in the study including genome accession numbers and multilocus sequence type. Supplementary table 1

## References

[1] Köser C.U., Holden M.T.G., Ellington M.J., Cartwright E.J.P., Brown N.M., Ogilvy-Stuart A.L. (2012). Rapid Whole-Genome Sequencing for Investigation of a Neonatal MRSA Outbreak. N Engl J Med.

[2] Harris S.R., Cartwright E.J., Török M.E., Holden M.T.G., Brown N.M., Ogilvy-Stuart A.L. (2013). Whole-genome sequencing for analysis of an outbreak of meticillin-resistant *Staphylococcus aureus*: a descriptive study. Lancet Infect Dis.

[3] Reuter S., Török M.E., Holden M.T., Reynolds R., Raven K.E., Blane B. (2016). Building a genomic framework for prospective MRSA surveillance in the United Kingdom and the Republic of Ireland. Genome Res.

[4] Coll F., Harrison E.M., Toleman M.S., Reuter S., Raven K.E., Blane B. (2017). Longitudinal genomic surveillance of MRSA in the UK reveals transmission patterns in hospitals and the community. Sci Transl Med.

[5] Chow A., Lim V.W., Khan A., Pettigrew K., Lye D.C.B., Kanagasabai K. (2017). MRSA Transmission Dynamics Among Interconnected Acute, Intermediate-Term, and Long-Term Healthcare Facilities in Singapore. Clin Infect Dis.

[6] Coll F., Raven K.E., Knight G.M., Blane B., Harrison E.M., Leek D. (2020). Definition of a genetic relatedness cutoff to exclude recent transmission of meticillin-resistant *Staphylococcus aureus*: a genomic epidemiology analysis. The Lancet Microbe.

[7] Talbot B.M., Jacko N.F., Petit R.A., Pegues D.A., Shumaker M.J., Read T.D. (2022). Unsuspected Clonal Spread of Methicillin-Resistant *Staphylococcus aureus* Causing Bloodstream Infections in Hospitalized Adults Detected Using Whole Genome Sequencing. Clin Infect Dis.

[8] Gordon N.C., Pichon B., Golubchik T., Wilson D.J., Paul J., Blanc D.S. (2017). Whole-Genome Sequencing Reveals the Contribution of Long-Term Carriers in *Staphylococcus aureus* Outbreak Investigation. J Clin Microbiol.

[9] Delmas J., Chacornac J.P., Robin F., Giammarinaro P., Talon R., Bonnet R. (2008). Evaluation of the Vitek 2 System with a Variety of Staphylococcus Species. J Clin Microbiol.

